# Characteristics, Challenges, and Support of Maryland's Direct Care Workers: Findings From a Statewide Agency Survey

**DOI:** 10.1093/geroni/igab046.835

**Published:** 2021-12-17

**Authors:** Chanee Fabius, Deirdre Johnston, Jennifer Wolff

**Affiliations:** 1 Johns Hopkins Bloomberg School of Public Health, Baltimore, Maryland, United States; 2 Johns Hopkins University School of Medicine, Baltimore, Maryland, United States

## Abstract

Direct care workers (e.g., personal care aides) are paid health care professionals who provide hands on assistance with daily activities to persons with disabilities in home, community, and institutional settings. Many workers are employed by direct care agencies, but little is known or understood about the organizational attributes of these agencies. We describe results from a mixed mode (postal mail, electronic, and telephone) survey of n=1112 residential care agency administrators in Maryland to assess organizational (e.g., size, supplemental services) and direct care worker (e.g., training) characteristics. Preliminary findings indicate that half of direct care agencies’ revenue comes from Medicaid and roughly 40% of clients are living with dementia. Administrators report challenges managing dementia-related behaviors (70%), communicating with persons living with dementia (63%) and interacting with family caregivers (63%). Findings from this work will inform the development of an organizational level intervention that targets training and support of direct care workers.

